# Maintaining Schooling for Children With Cancer During and Post Treatment: Parents’ Perspectives of a Theory-Based Program

**DOI:** 10.5334/cie.24

**Published:** 2021-03-15

**Authors:** Stella Delloso, Anne Gannoni, Rachel M. Roberts

**Affiliations:** 1 University of Adelaide, AU; 2 Women’s and Children’s Health Network, AU

**Keywords:** childhood cancer, oncology, schooling, school re-entry, perceptions, education

## Abstract

This study explored parents’ perceptions of a hospital-based schooling intervention for children with cancer. A qualitative design using semi-structured interviews was employed. Participants were nine parents whose children had participated in the program. Parents participated in semi-structured interviews, which were transcribed verbatim and analysed thematically. Five major themes were identified: experiences with program components, the bigger picture of the program, communication, a burden of responsibility for parents, and perceived impacts of cancer on schooling. Results showed that the parents valued the focus upon schooling and viewed several aspects as being beneficial. Challenges for parents included a lack of communication and individualized planning, and a burden of responsibility. Findings suggest that schooling is important to families and should be a fundamental psychosocial consideration of service providers. The schooling-related needs of parents should also be addressed.

With improved outcomes for most childhood cancers, children diagnosed with cancer now have increased opportunity to re-engage in school. Although recommendations for supporting the schooling of children with cancer exist (Lum et al., 2017; Thompson et al., 2015), to date few hospital-based interventions have been reported; thus, little evidence is available to guide clinical practice.

The Oncology Education Program (OEP) is a hospital-based schooling intervention developed at a children’s hospital in Australia. Delivered since 2015, the program appears unique in its interdisciplinary, theory-driven, and systemic approach to service delivery. This paper describes parents’ views about their and their child’s experiences of participating in the program.

## The Impact of Cancer on Schooling

Cancer and its treatment are likely to impact children’s school adjustment (e.g., engagement, academic and social functioning) via three main illness-related pathways: impaired school attendance, physical and cognitive effects, and psychosocial effects (Perry & Weinstein, 1998; Sexson & Madan-Swain, 1993). As a result, compared to children without cancer, children with cancer have a higher likelihood of grade repetition (Barrera et al., 2005; Bonneau et al., 2011; Roberts et al., 2014), having fewer close friends (Barrera et al., 2005), and poorer academic outcomes (Barrera et al., 2005) affecting their educational attainment and employment (Maule et al., 2017; Mitby et al., 2003). As school provides respite from treatment, returning to school early and attending regularly likely serves a normalizing function that may facilitate illness adjustment (Mavrides & Pao, 2014; Prevatt et al., 2000).

## Schooling Interventions

Thompson et al. (2015) developed a psychosocial standard of pediatric oncology care, recommending universal schooling, including school re-entry, utilizing timely and supported approaches. This was supported by a subsequent meta-analysis (Helms et al., 2016), which found that schooling intervention for children with cancer was associated with positive effects, including enhanced academic achievement and lowered depression levels for the child with cancer, increased knowledge among peers and a more positive classroom attitude towards the child with cancer. However, according to Lum et al. (2017), in the Australian context, no interventions exist that address the standards outlined by Thompson et al. (2015). To fill this gap, Lum and colleagues proposed a set of baseline requirements for Australian schooling interventions (see ***Table 1***).

**Table 1:** Baseline Requirements of the Proposed Australian School Re-Entry Guidelines (From Lum et al., 2017).


HEALTH AND EDUCATION PROFESSIONALS CARING FOR CHILDREN NEED TO …

Provide a continuing, flexible education program in hospital or home

Assign a pediatric oncology team member as the hospital-based liaison officer

Assign a school team member as the school-based liaison officer

Establish a collaborative learning support team to regularly meet, involving family, school and hospital personnel

Develop an individualised education plan that is available to all school and hospital staff

Develop an individualised health plan that is available to all school and hospital staff

Transition the child to school as soon as possible

Maintain communication between school, family and hospital

Provide resources for teachers to understand the illness, its educational implications and how they can be managed at school

Educate classmates about cancer and its implications

Monitor academic functioning annually throughout school enrolment

Monitor psychosocial well-being annually throughout school enrolment

Identify and monitor high-risk students (e.g., CNS treatment)

Give special consideration to transition periods (e.g., progressing from primary to high school)

Provide information regarding legally bound educational support

Have in place, if necessary, a special palliative care plan for the student, their peers and teachers


While general support for schooling interventions exists, descriptions of them, and hence evaluation, seem almost non-existent. Further, the interventions that have been described appear to have minimal theoretical underpinning and limited evaluation of separate components (Helms et al., 2016; Lum et al., 2017; Prevatt et al., 2000; Thompson et al., 2015; Tollit et al., 2015; Vance & Eiser, 2002). The lack of consistency in program target (e.g., child, teachers, peers), aims, components, and outcome measures across studies is problematic for synthesizing the literature. Consequently, despite growing recognition of the need to incorporate schooling intervention into childhood cancer care, no evidence-based approaches to guide practice are available (Libman et al., 2017; Lum et al., 2017).

## The Oncology Education Program (OEP)

The OEP was developed by clinicians to address a service provision gap and, particularly, in response to an earlier study (Roberts et al., 2014) that found a significant proportion of children treated for cancer at an Australian children’s hospital had repeated a grade. Briefly, the OEP prioritises schooling during treatment, facilitates school engagement, and promotes academic and social outcomes. This includes supporting children to maintain positive links with their enrolled school and to return to school as soon as medically able.

**Figure 1:**
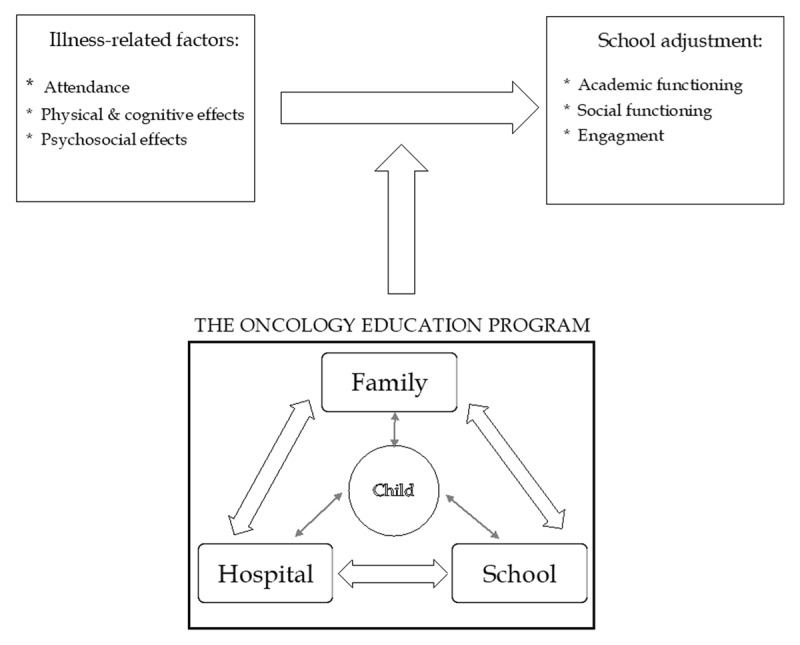
The OEP theoretical model.

While many individual illness-related aspects of cancer that impact schooling are not readily modifiable, a socioecological framework provides an alternative pathway for targeting schooling, via the systems surrounding the child. Bronfenbrenner (1994) proposes that a child’s development is best considered in the context of the environments in which they function, acknowledging the reciprocal relationships between the developing child and different levels of their environment, including settings that they interact with directly (microsystems), and the linkages between these settings (the mesosystem). For a child with a chronic illness, the most influential settings are likely to be family, the hospital, and school (Power, 2002; Brown, 2002). Based on Bronfenbrenner’s (1994) influential model and child chronic illness adjustment models (e.g., Risk and Resilience Model, Wallander & Varni, 1998; the Transactional Stress and Coping Model, Thompson & Gustafson, 2009), the OEP’s theoretical model (see ***Figure 1***), proposes that supporting the systems surrounding the child and improving collaboration between these systems will enhance schooling experiences and outcomes. While such a socio-ecological theoretical approach to school intervention has been suggested (Brown, 2002; Power et al., 2003) and underpins the recommendations by Lum et al. (2017), it has not been well explored.

**Figure 2:**
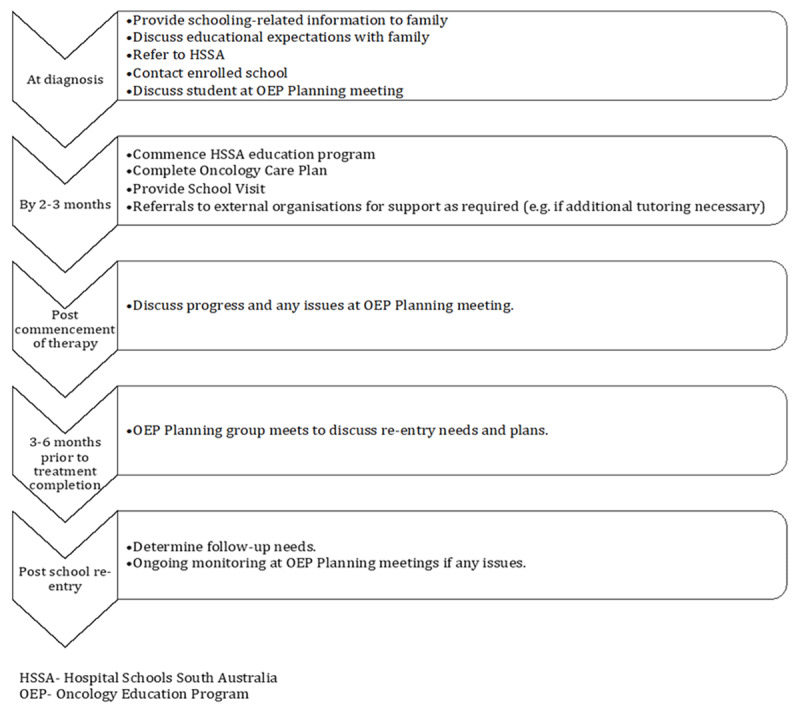
The OEP pathway.

The educational program within the OEP is delivered by Hospital Schools South Australia (HSSA), based at the children’s hospital, and consists of several flexible, individualized components designed to address both children’s educational and social needs, in hospital, at home, and at school. An additional interdisciplinary component is a school visit, attended by a school liaison nurse and HSSA representative (see ***Table 2***). Interdisciplinary planning and review groups have also been formed across education, medical, and psychology departments and a program pathway has been developed (see ***Figure 2***). The interdisciplinary nature of the program is integral to its systemic approach and the introduction of system-level change within the hospital, such as increasing the priority given to schooling within a medical setting.

**Table 2:** OEP Components.


OEP COMPONENT	DESCRIPTION

Face-to-face teaching	Aims to engage students in targeted learning tasks to assist in their progress while unable to attend their enrolled school. Provided in the HSSA classroom and on the wards (at the bedside or in the playroom).

Learning online	WebEx used as the online platform to participate in face-to-face HSSA teaching sessions, individually and/or in groups. WebEx can also allow students to connect with their class via video conferencing. Through learning online, students are able to participate in lessons and/or engage with peers socially.

Learning@Home	Aims to continue and consolidate students’ learning progress while at home and not able to attend their enrolled school. Learning packs are sent home in the mail. Maintains students’ connection with their key teacher from HSSA and normalises their day by engaging in learning tasks.

Connecting Kids	Aims to maintain students’ relationships and connections with school friends and encourage engagement in fun writing activities. Maintaining connection likely to ease the transition back to school. Adapted to suit different ages and interests; – For preschool and junior primary students, connection with friends is facilitated through two identical soft toys retelling events to each other. – For primary students, a scarf or jumper of the child’s favourite sporting team is often used. – Middle and senior secondary students typically prefer to use their own school’s IT platform, personal emails, and phone texting to stay connected.

Waiting room visits	Face-to-face teaching and/or contact and support from HSSA teachers provided while children are waiting for treatment or appointments.

School visit	A school liaison nurse and HSSA representative visit the student’s enrolled school to provide school staff with disease- and treatment-related information. An information pack is provided, including an oncology patient care plan, a letter template for informing the school community about the risk to the student of infectious disease, relevant community support services, tutoring options and hospital contact information.


*Note*: HSSA = Hospital Schools South Australia.

## Objectives

This study aimed to better understand the experience of participating in the OEP and, based on parents’ views about the program, including its components and its impact, to inform future program development. Further, given gaps in the literature, the study sought to gain a better understanding of parents’ perspectives on the school adjustment processes for children who have participated in the program. Parents’ experiences were investigated using a qualitative inductive approach, allowing for the exploration of lived experiences of parents whose children had been treated for cancer.

## Method

### Recruitment and Sampling

Parents of children with cancer were recruited by nursing staff. Parents were eligible to participate if their child was of school or kindergarten age during treatment (3–18 years), had received treatment for cancer at the participating children’s hospital between May 2015 and March 2018, and was in the maintenance phase of treatment or had completed treatment.

Between September 2018 and January 2019, all parents who met eligibility criteria (*N* = 106) were invited to participate. Initially, nursing staff recruited through face-to-face contact, but since only 12% of potential participants (*N* = 13) had been approached by nursing staff after four months, the protocol was amended, whereby the remaining participants (*N* = 93) could be contacted by nursing staff by mail. Families that returned an expression of interest were then contacted by the primary researcher. Non-identifiable demographic data was collected for families who declined to participate.

### Data Collection

A semi-structured interview guide was developed as a means of gaining insight into the experience of participating in the OEP and schooling with cancer. The guide included open-ended questions exploring program components, facilitators, impacts, barriers, and gaps. Examples include “What is your understanding of the Oncology Education Program?” and “Could you tell me more about what it was like when your child returned to school after treatment?” (See Appendix A for the complete interview guides for parents.) Interviews were conducted by the principal researcher, a provisionally registered psychologist, either face-to-face at the children’s hospital or by telephone, depending on the parents’ preference, and audio recorded. Interviews lasted an average of 47 minutes (*SD* = 9.2).

Demographic and medical variables that may affect the schooling experience (e.g., child’s gender, type of cancer, age at diagnosis, phase of treatment) were collected via parent-report and supplemented using hospital medical records. In addition, HSSA program participation was collected to provide a measure of program engagement.

### Data Analysis

Interview data were analysed by the first author using Braun and Clarke’s six-step method of thematic analysis (2006, 2013), taking a data-driven, inductive approach to allow unexpected themes in the data to be identified, with the most dominant and important themes for the participants reported.

First, interview data were transcribed verbatim, followed by familiarization of the data by reading each transcript multiple times. Using a semantic approach, initial codes were generated, and once all data had been coded, these initial codes were synthesized into potential subthemes and themes. Codes and themes were discussed and reviewed by the research team (the study authors), whereupon the themes were defined, refined further, as appropriate, and named. This cross-checking with the team contributed to the clarity and trustworthiness of the themes. During analysis of the final three transcripts, no new themes emerged, suggesting that data saturation had been reached. Finally, compelling extracts were selected to illustrate themes.

## Results

### Participant Characteristics

Of 106 families approached to participate, 8 mothers and 1 father from 8 families completed an interview (response rate 7.6%). Demographic information is reported in ***Table 3***. To protect anonymity, variables such as diagnosis have not been linked with individuals.

**Table 3:** Demographic Information on Children With Cancer.


PARENT	CHILD	GENDER	AGE (YEARS) AND SCHOOL GRADE AT INTERVIEW	AGE (YEARS) AND SCHOOL GRADE AT DIAGNOSIS	AMOUNT OF SCHOOLING MISSED (APPROX.)

Parent 1 (Mother)	Child 1	F	6(1)	5(Rec)	>6 months

Parent 2 (Mother)	Child 2	F	9(3)	6(Rec)	>6 months

Parent 3 (Mother)	Child 3	F	9(3)	7(1)	>6 months

Parent 4 (Mother)	Child 4	M	8(3)	7(2)	>6 months

Parent 5 (Mother)	Child 5	M	6(1)	3 Relapse at age 6 (Rec)	3–4 months

Parent 6 (Father)	Child 6	M	8(3)	5 (Rec)	>6 months

Parent 7 (Mother)	Child 6				

Parent 8 (Mother)	Child 8	M	6(2)	1 Relapse at age 5 (Rec)	1–2 months

Parent 9 (Mother)	Child 9	M	15(10)	12(6)	>6 months


*Note*: Rec = Reception (first grade of schooling in South Australia).

Acute lymphoblastic leukemia (ALL) was the most common diagnosis (50%). This is representative of all eligible participants, of whom 43.9% had a child diagnosed with ALL. Other diagnoses included Wilm’s tumor, Hodgkin’s lymphoma, acute myeloid leukemia and spinal cord tumor. Child gender (62.5% male) was representative of the total eligible sample (61% male). Seventy-five percent of children in the study attended a public school at the time of diagnosis. The average age of children with cancer in participating families was 8.4 years (*SD* = 3.0), and the average age of diagnosis was 6.5 years (*SD* = 2.5). There was an overrepresentation of families with a primary school-aged child and rural/remote families, compared to all eligible families (see ***Table 4***). Each child’s engagements with each component of the OEP are displayed in ***Table 5***.

**Table 4:** School Age Group and Rural/Remote Status of Participating Families Compared to All Eligible Participants.


	STUDY SAMPLE (%)	ALL ELIGIBLE FAMILIES (%)

Preschool age (<5yrs)	12.5	33

Primary school age (5–12 yrs)	75	47

High school (13–18 yrs)	12.5	20

Rural/remote	50	16


**Table 5:** Engagements With Each Component of the OEP.


	FACE-TO-FACE		CONNECTING KIDS	LEARNING @HOME	WAITING ROOM	LEARNING ONLINE	SCHOOL VISIT	TOTAL ENGAGEMENTS

	CLASSROOM*	WARD						

Child 1	1	39	Y	6	8	1	Y	57

Child 2	1	41	N	1	0	0	N	43

Child 3	4	39	Y	3	4	46	Y	98

Child 4	0	25	Y	3	2	5	Y	37

Child 5	0	2	Y	0	1	0	Y	5

Child 6	0	39	Y	2	6	0	N	48

Child 8	0	0	N	0	0	0	Y	1

Child 9	0	45	N	0	6	8	N	59

**AVERAGE NUMBER OF ENGAGEMENTS PER CHILD**	**0.75**	**28.75**		**1.88**	**3.38**	**7.50**		**43.50**

**(*M, SD*)**	**(1.92)**	**(18.07)**		**(2.10)**	**(3.07)**	**(15.84)**		**(31.04)**


*Note*: Classroom = Hospital Schools SA classroom, located on-site at the children’s hospital.

### Themes

Five major themes and nine subthemes were identified (***Table 6***).

**Table 6:** Themes and Subthemes.


1. Experiences with program components

Ways of learning

Connecting kids

School visit

Other sources of academic and social support

2. The bigger picture of the program

Parent understanding

The message – Schooling is important

3. Communication – Not proper three-way

4. A burden of responsibility for parents

The school takes a backseat

What’s the plan?

A lack of resources

5. Perceived impacts of cancer on schooling


#### Theme 1: Experiences With the Program Components

##### Ways of Learning

Parents discussed a range of ways of learning that supported their children’s learning, including face-to-face teaching, waiting room visits, education materials being provided to them at home, and learning online.

All families except one participated in face-to-face teaching. These parents valued the maintenance of schooling in the hospital, describing it as normalizing and a positive distraction. Most parents commented upon teachers’ flexibility and children’s enjoyment of the programme and their connection with teachers. One parent identified face-to-face teaching as instrumental in preventing grade repetition.

*The school was pretty quick to decide she’d have to repeat Reception [first year of formal schooling in South Australia], because they said she’d missed too much school … and so they [HSSA] visited her and helped … getting her up to a level that the school wanted*. (Parent 2)

However, many parents expressed unmet expectations related to face-to-face teaching, including more frequent visits, more dependable scheduling, and individualised programming that better addressed their perceived academic needs for their child.

Parents viewed the waiting room visits as a positive distraction but did not identify any educational value attached to them.

*Yes, it was a nice distraction in the waiting room potentially for a little bit, but there was nothing probably hugely educational*. (Parent 6)

Parents reported that receiving Learning@Home packages in the mail was a source of excitement and distraction, but some felt they were not frequent enough.

*It was a fun way of learning, kept her in the habit*. (Parent 2)

No families had the opportunity to use WebEx with their own school, and only three participated in sessions with HSSA, but most parents responded that they would have liked both opportunities. The families that had access to learning online reported that it was a useful option to flexibly continue schooling at home.

*… to actually have to do something at home when he was quite well but still couldn’t go out, that was valuable*. (Parent 9)

##### Connecting Kids

The purpose of this component appeared to be well understood and appreciated by parents. For some, Connecting Kids was reported as the only contact the child had with his or her class while absent from school. All families had a name for their soft toy, and it was discussed fondly.

*… we got text messages from families taking [stuffed toy] places and it sat at her desk … The teacher said they talked about her [Child 3] nearly every day at school, that she still felt like part of the class*. (Parent 3)

##### School Visit

The school visit was one of the most discussed components. It was viewed positively as a much-needed sharing of information from hospital to school.

*… there were definitely times when it was difficult for him to go, and I think the fact that his teacher had met with professionals helped her … it provided her with support*. (Parent 8)

However, despite the program aim of universal school visits, not all participating families in this study received one. Of those who did, several felt that parents should have been included. Most parents also expressed issues with the timing of the visit and the need for the visit to be repeated at key transition times, such a transitioning back to school and into new school years. This need was discussed at length, signifying a gap that had been challenging for parents.

*It [the school visit] was soon after diagnosis, and [Child 4] wasn’t back at school for a year … schools need someone to go out and say here’s how you support this child coming back to school … it was too early in [Child 4’s] case* … (Parent 4)

##### Other Sources of Academic and Social Support

Parents reported that their child’s schooling adjustment was assisted by the wider community. Social contact during treatment was often arranged between parents or occurred in the context of sporting clubs. More formal schooling support included tutoring through the Childhood Cancer Association (CCA) and the Ronald McDonald Learning Program, counselling from CCA, and classroom presentations from Camp Quality (organization that provides support for children with cancer). These extra supports were usually organized by parents themselves and were viewed positively.

*… we were offered tutoring through CCA … and it alleviated that stress that he would fall behind the other children after having a year at home … we highly recommend that*. (Parent 5)

#### Theme 2: The Bigger Picture of the OEP

##### Parent Understanding

When asked to describe their understanding of the program, most parents reported confusion. Only one parent seemed aware of the comprehensive nature of the program model; the others tended to focus more narrowly upon access to HSSA activities.

*Well, I don’t really have much understanding, I didn’t really have that much information … I know that when we came in the teacher would sometimes come and see us … I don’t know anything really about what their goals are or anything like that*. (Parent 9)

##### The Message – Schooling Is Important

Despite minimal understanding of the program model, and some issues with certain elements of program delivery, all families appeared to agree with and value the premise of the program, that schooling should be an important part of their child’s cancer care. Support from the hospital system for the notion that schooling is important appeared the main benefit of the program for parents.

While the priority given to schooling fluctuated for families and over time, parents described several benefits of maintaining schooling. These included academic, emotional, social, routine, normalizing, and life-affirming aspects.

*It was important … it was important to have routine and it was important that he … didn’t miss a whole lot of school. Routine … is really good for you and the distraction of school was important, and also just him getting an education*. (Parent 8)

#### Theme 3: Communication – Not Proper Three-Way

Although parents desired a high level of three-way communication between family, school, and hospital, they did not think that this occurred.

*I just would’ve liked that to be proper three way … sometimes I wasn’t included in that hospital to school communication and it would’ve helped me to know what’s going on … It became tricky because I was either running it or excluded from it*. (Parent 3)

#### Theme 4: A Burden of Responsibility for Parents

All parents, except one whose child had missed little school, discussed feeling a significant burden of responsibility related to their child’s schooling. Examples of this responsibility included driving the communication between the appropriate parties, providing educational support, monitoring progress, and advocating for their child. Parents described this extra burden as exhausting and stressful, especially as they navigated their child’s health care requirements.

*I feel like I always had to drive it, and I was exhausted. I’d had enough working out what meds she [Child 3] was on, which protocol she was on and whether … she was alright and whether my son [sibling] was alright and who was looking after him, and who was doing what and then to add – you know –“Can you send us this work?” each week*. (Parent 3)

Three main factors related to schooling appeared to contribute to this burden of responsibility: (a) lack of responsibility from the child’s school, (a) lack of three-way communication, and (c) lack of resources and support once the child was back at school.

#### The School Takes a Back Seat

A common experience among families was a lack of knowledge and initiative from their schools. Most parents reported that their schools did not appear to know what to do when told about their child’s diagnosis. While some parents reported a general sense of supportiveness from their school, little practical help was offered. Most parents reported that they had to be the ones driving all academic-related communication with their school.

*What I would have liked was a liaison appointed, someone who contacted us rather than me going in there all the time … To me it was hard to always be the one to have to go and contact them*. (Parent 4)

#### What’s the Plan?

All parents expressed a persistent concern about their child’s educational progress, both during treatment and when back at school. Most parents worried about whether their child’s learning was being planned or monitored, and whether it was “enough.”

*It was much more that gap of when we were at home, what am I meant to be doing with him, how do I teach him? … we did have to reach out for that information rather than here’s your plan sort of thing*. (Parent 7)

#### A Lack of Resources and Support for Kids With Cancer at School

Several parents felt that their school had minimal resources to support their child, particularly during the return transition to school. Parents appeared to be the stopgap and would stay and support their child in the classroom, often for months.

#### Theme 5: Impacts of Cancer on Schooling

Parents discussed ways in which cancer impacted their child’s schooling, including prolonged absence and physical, emotional, behavioural, social, and academic impacts. Concerns appeared to be greatest in amount and intensity during the transition back to school. While most parents discussed some anxiety related to the impact on their child of treatment-related changes, many also remarked on their child’s resilience and on peers’ preparation and acceptance of these changes.

*She handled it really well when kids would say things like “your face has changed,” because the steroids would make her face puff up, and she would go, “Oh, it’s just my medicine.” She had that inner confidence, I suppose*. (Parent 3)

Typically, absences of more than six months meant that parents often reported a sense of “missing out” felt by both themselves and their child relating to school experiences, milestones, and time with friends.

Some parents also identified problematic changes in both their child’s approach to learning and their teacher’s approach to teaching them.

*… it became that funny thing where [the child’s teacher] sort of admired her too much, she’s so brave, she’s amazing, I was like, well, she still has to do her spelling!… it changed the relationship and changed the expectation, and her ability to follow instructions changed because she thought everything’s optional*. (Parent 3)

Few explicit links were made between the OEP and schooling outcomes. One parent attributed support from HSSA as key in preventing grade repetition. Another parent reported that the academic maintenance and school preparation provided by the program were generally valuable.

*I think they did a great job, anything to do with prepping the school socially for her to come back was done really well … the hospital academic side of things was fantastic, it was targeted, it was flexible … and the school did a great job socially in that she could walk in there with no hair and no one commented*. (Parent 3)

Despite some challenges, no child in the study ended up repeating a grade, and all were described by their parents as now relatively well adjusted and enjoying school;

*When he went back to school, he was so motivated and so excited to get back … I think there’s no lasting impact of him having those six terms [1½ academic years] off school*. (Parent 9)

## Discussion

While schooling with cancer poses challenges, maintenance of schooling provides several potential benefits. This study explored parents’ experiences of being involved in a hospital-based schooling intervention. Parents provided rich narratives reflected in five themes and nine subthemes. All parents agreed with and valued the central program message that schooling is an essential component of their child’s wellbeing, with routine and normality being significant benefits of the program. While parents focused on child-related benefits, a focus beyond cancer appeared to be normalizing and life-affirming for parents themselves, a need noted in the literature on parents with children with serious illness (McKevitt et al., 2018; Prevatt et al., 2000). Schooling offered parents brief respites from the caring role and an opportunity to view their child as a student rather than a patient, participating in developmentally typical, future-oriented activities.

The results of the study suggest that the family and health systems have the same overall schooling-related goals. This was fundamental to ascertain, as while parental support is key, parents’ priorities and values cannot be assumed to align with those of health professionals (Woodend et al., 1997). The reasons why parents viewed schooling as important varied and may indicate differences in their underlying values and motivations and, to some extent, explain diversity in their appraisal of the OEP.

Interesting, parents perceived the school system to be the least supportive of the notion that schooling remains important after a cancer diagnosis. Most parents reported that while sympathetic, they perceived that the enrolled school “took a back seat,” and did not initiate contact to maintain, monitor, or plan schooling. The school stepping back after cancer diagnosis has been noted previously (Marshall, 2017) despite Australian law stipulating that education remains the enrolled school’s responsibility (Department of Education, Science and Training, 2005; Lum et al., 2017) and the fact that the student-teacher relationship is one of the most powerful predictors of a student’s sense of belonging (Allen et al., 2018). Student-class relationships are also important in the context of students with chronic medical conditions experiencing lower levels of school belonging (Kirkpatrick, 2020) and was reflected in the theme Connecting Kids, where parents valued the efforts made to support their child’s connection to their class. Research suggests that teachers are not well informed about managing chronic illness and may feel overwhelmed and reluctant to intervene (Brown, 2002; Moore et al., 2009; Prevatt et al., 2000; Wilkie, 2012). Also consistent with the literature, parents reported changes in teaching style such as lowered expectations and excessive admiration upon their child’s cancer diagnosis (Brown, 2002; Rynard et al., 1998). Overall, the school system was perceived as not meeting the needs of parents and, therefore, may require additional support to better understand its role and address challenges.

The perception that no one was monitoring their child’s schooling progress and that no formal plan had been developed was linked with significant burden for parents. It may be that there remains confusion across systems regarding academic expectations for children with cancer and who is responsible for producing such a plan. Such system-level confusion is reported in the literature (Moore et al., 2009) and relates to Bronfenbrenner’s (1994) socioecological framework, which can be used to explain system-level influences upon a child and the support needed to best adjust to and ensure continuity following significant events and transitions (relating to Bronfenbrenner’s chronosystem concept – the idea that time is another level to be considered). Alternatively, it may be that children’s schooling is being planned and monitored, but that these details are not being communicated with parents, which is feasible given that the program pathway aims, within three months from diagnosis, to make contact with the enrolled school, complete a school visit, and make referrals to external agencies for additional supports. The meaningful inclusion of students in educational planning, consistent with Bronfenbrenner’s concept of molar activity has been described (see Capurso, 2015), and parents in the current study noted the need to extend this to include the meaningful involvement of parents. Regardless of which interpretation is the more accurate, parents reported being unaware that a learning plan had been developed and felt that this burden fell to them, despite feeling ill equipped to assume this responsibility. The creation of an individualized learning plan, available to all school and hospital staff, is a key recommendation proposed by Lum et al. (2017). This study supports the inclusion of parents in this recommendation.

Little research has explored the role that schooling-related demands play in the overall caregiving burden experienced by parents. After a diagnosis of their child’s cancer, parents manage several aspects of their child’s illness, including educational needs. As parental stress and exhaustion can have negative sequelae for both parent and child (Wakefield et al., 2009), health and education professionals must also be mindful of the support that parents need.

The concern driving parents to desire more rigorous academic planning and programming is particularly interesting to explore. Specifically, it is noteworthy that concerns about “keeping up” were salient, given that many children were in Reception during their intensive treatment phase. However, it seems that more complex worries lay beneath underlying schooling concerns – “Will my child be able to return to a normal life post-cancer?” Thus, a tendency to link current school progress to projected life outcomes may explain why parental worries about progress were prominent even when academic expectations were low. This study suggests that parents are driven to minimize the impact of cancer and are attuned to monitoring for gaps. Such increased parental involvement in education is likely to create parental needs that are more intense than schools are accustomed to.

It appears that underpinning several parental perceptions in this study is a lack of effective communication between systems. Bronfenbrenner’s model described the importance of the mesosystem for connecting the microsystems surrounding the child (Bronfenbrenner, 1994) and the importance of these connections is also noted in our model presented in ***Figure 1***. While effective three-way communication between the family, health and school systems is at the core of successful school support (Department of Education, Science and Training, 2005; Lum et al., 2017; Pini et al., 2011; Thompson et al., 2015), parents in this study expressed a need for improved communication. Communication gaps could account for, or mitigate, several program-related gaps and barriers for parents. The findings of this study suggest that while parent contact may be limited in an attempt not to overburden them, this lack of involvement paradoxically increases the burden parents feel.

Furthermore, it is of concern that parents reported being the stop gap for within-classroom support, often for many weeks. The back-to-school transition and schooling at home have been identified as phases associated with increased stress (McLoone et al., 2011; Wakefield et al., 2009), and again suggests that more support for families and communication across systems is necessary. This finding is also consistent with a recent Australian study showing that most students with chronic health conditions missed more school time than the support service could respond to in terms of making up for lost learning, with many students missing over 40 days of school in a year but receiving teaching support equivalent to less than 2 days of school (Cave et al., 2020).

A pediatric oncology team member acting as school liaison is a key component of re-entry guidelines proposed by Lum et al. (2017). The nature of the school liaison role has varied widely in the literature (Bruce et al., 2012; Gannoni & Shute, 2010; McLoone et al., 2011). In the present study, the role was defined by parents as providing medical information at the school visit only rather than ongoing support. Consistent with Bruce et al. (2012) and McLoone et al. (2011). parents identified the school liaison as a possible advocate and suggested that the role should be expanded to include ongoing support and advocacy. In the literature, a school liaison model involving ongoing support has been linked to high satisfaction and perceived support and better academic outcomes (Northman et al., 2014).

Parents described several impacts of cancer on their child’s school adjustment, the greatest impact being during the transition back to school. Overall, however, parents painted a positive picture of their child’s school re-adjustment. Consistent with Rynard et al. (1998), parents reported few ongoing academic or social concerns. In contrast to the key study by Roberts et al. (2014) where almost a quarter of students repeated a grade, no students in the current study had needed to. While the positive outcomes reported by parents cannot be attributed directly to the OEP and this is only a small, select sample, this suggests that the experience of schooling has been largely positive for these children at least.

Recruitment for this study was difficult. For example, ethics requirements that parents could not be approached directly by the researchers created considerable work for hospital staff. Strategies employed to address this obstacle included (a) the researcher remaining available on site, (b) regular meetings with staff, and (c) contacting eligible families by mail. Furthermore, with low participation and the need for timely completion of the study, it became necessary to reduce the scale and scope of the study. While it is desirable for future research to triangulate data from a number of sources (e.g., teachers, parents, children), across systems (e.g., health, education), and broader data sources (e.g., both qualitative and quantitative data), this study highlights barriers to evaluating programs within a clinical setting, such as caregiver treatment and research fatigue (Clark, 2008; Pagano-Therrien & Sullivan-Bolyai, 2017). Families of children with chronic illness may be particularly susceptible to these barriers. A further issue with the need for hospital staff to approach potential participants is that parents with more negative experiences may have been reluctant to respond, whereas an approach from a researcher from an independent research organisation (the university) might have been more attractive to them.

It is recognised that the experiences of this sample of parents may not be generalisable to all OEP participants. These parents were motivated to participate and may have placed greater focus on education. Thus, recent findings showed that high-achieving students with chronic health conditions were more likely to opt into receiving support from special education support services (Cave et al., 2020). Experiences also pertain largely to the early schooling years. Nevertheless, this study provides new information about the experiences of parents with a comprehensive hospital-based schooling intervention. Despite low participation, participation fell within recommendations for a small-scale qualitative study (Braun & Clark, 2013), and saturation of all key themes was reached, indicating that important commonalities of participating in the OEP were established.

### Implications

To our knowledge, this is the first Australian hospital-based schooling intervention reported in the literature. The theory-based intervention model presented addresses many recommendations proposed by Lum et al. (2017) (see ***Table 7***), and responds to criticism that the separate components of school interventions have not been well described (Helms et al., 2016; Lum et al., 2017; Thomson et al., 2015). Findings were consistent with the proposed theoretical model; that is, parents felt that supporting systems surrounding their child, and improving collaboration between these systems, were key to enhancing schooling experiences and outcomes. Parents valued the program’s focus on schooling and were satisfied with their child’s eventual school adjustment. However, several areas for improvement and program development were also highlighted, including (a) more frequent and structured communication, (b) collaborative development of an individualized schooling plan, (c) expansion of the school liaison role, (d) additional support for families and schools during transition periods and beyond, (e) greater support for parents, and (f) greater parental awareness about the program itself.

**Table 7:** Recommendations Addressed by the OEP (Adapted From Lum et al., 2017).


	HEALTH AND EDUCATION PROFESSIONALS CARING FOR CHILDREN NEED TO …

Yes	Provide a continuing, flexible education program in hospital or home

Yes	Assign a pediatric oncology team member as the hospital-based liaison officer

Partially	Assign a school team member as the school-based liaison officer

Yes	Establish a collaborative learning support team to regularly meet, involving family, school, and hospital personnel

Partially	Develop an individualised education plan that is available to all school and hospital staff

Yes	Develop an individualised health plan that is available to all school and hospital staff

Yes	Transition the child to school as soon as possible

Yes	Maintain communication between school, family, and hospital

Yes	Provide resources for teachers to understand the illness, its educational implications and how they can be managed at school

No	Educate classmates about cancer and its implications

Yes	Monitor academic functioning annually throughout school enrolment

Yes	Monitor psychosocial well-being annually throughout school enrolment

Yes	Identify and monitor high-risk students (e.g., CNS treatment)

Partially	Give special consideration to transition periods (e.g., progressing from primary to high school)

No	Provide information regarding legally bound educational support

Yes	Have in place, if necessary, a special palliative care plan for students, their peers, and teachers


*Note*: Yes = addressed by the OEP, Partially = partially addressed by the OEP, No = not addressed by the OEP.

### Conclusion

Limited research has explored the experiences of schooling intervention for families with a child with cancer. This study contributes to our knowledge about schooling with cancer and the experience of participating in a hospital-based schooling intervention. Following Bronfenbrenner’s multi-layered ecological systems model, a theoretical framework was developed to examine the effects of system-level schooling intervention after a diagnosis of cancer.

Findings suggest there are modifiable, system-level influences that moderate schooling experiences and outcomes for families that can be addressed by a hospital-based schooling intervention. Parents described significant stress involved in navigating their child’s schooling, and their experiences inform several recommendations for practice, including increased three-way communication and greater support for parents. Overall, however, parents valued the support they received from the hospital system in maintaining their child’s schooling and perceived minimal impacts of cancer upon their child’s schooling trajectory. While further research is necessary to evaluate its effectiveness, the study provides preliminary support for this hospital-based model of schooling intervention. In addition to school being an important developmental context, parents believe that maintenance of schooling benefits children with cancer and their families. Although this model relates to children receiving cancer treatment, the approaches described here are relevant to supporting all children’s education where there are restrictions to access to typical education (e.g., other illness groups, during pandemics), and future work, therefore, could consider this further.

## Additional File

The additional file for this article can be found as follows:

10.5334/cie.24.s1 Appendix A. Interview Guides.
