# Supplementary material for: Maintaining Schooling for Children With Cancer During and Post Treatment: Parents’ Perspectives of a Theory-Based Program
Source: Contin Educ. 2021 Mar 15;2(1):26–41. doi: 10.5334/cie.24 (PMC11104355; doi:10.5334/cie.24)
Supplement: Appendix A. — Interview Guides. [file cie-2-1-24-s1.pdf]

Appendix A Interview Guides

INTERVIEW GUIDE – PARENTS

(To be tailored according to stage of treatment and survey responses)

SCHOOLING AT DIAGNOSIS

1. **I'll start with the time of diagnosis, during the early stages, on finding out your child had cancer, did things at school change straight away?**
   - How?
   - What was the reaction from school in the early stages?
   - Who at school did you tell?
   - How did your child cope with school at that time (esp. with reactions from peers, etc.)?
   - Did you have any guidance?

SCHOOLING DURING INTENSIVE TREATMENT

2. **Then your child had a period of intensive treatment? Could you tell me about schooling during this period?**
   - How much time do you think that your child missed during this time?

   - Was the time missed mainly for hospitalisation or other reasons, too?
   - Did you have any contact with your child's school during this time (extended absence)?
   - Did your child have any contact with peers/friends?
   - How did your child feel about missing school?

ONCOLOGY EDUCATION PROGRAM (discuss in relation to survey responses)

3. **Could you tell me about whether schooling was discussed during intensive treatment period?**
   - Did you receive any information from the hospital about how your child's education would be continued during treatment? Did you find this helpful?
   - How was it initiated?
   - What was the reaction from your child's school?

4. **What is your understanding of the OEP at the [children's hospital]?**

5. **You have indicated on your survey that your child participated in _____ (insert components), could you tell me a little more about your experience with each of these parts of the program?**
   - Prompt for each component (except for school visit)

6. **What do you think was your child's experience of these parts of the program?**
   - Was the child enthusiastic/ interested/ engaged?

7. **Do you think that the services provided by the hospital met the schooling needs of your child and you at the time?**
   - Any needs that weren't met?

8. **What, if anything, did you find most helpful about the program?**

9. **What, if anything, did you find least helpful about the program?**

10. **Do you think your child had about the right amount of opportunities to participate in these programs?**
    - Would you have liked to participate more/ less?
    - Were there any barriers/obstacles to participation?

11. **How much contact with your child's school did you have during this period?**

12. **How much contact did your child have with peers/ friends during this period?**

<u>TIME OF RE-ENTRY TO SCHOOL</u>

13. **Then there was a point at which your child's treatment entered a maintenance phase and you were told your child was medically able to go back to school. How did you feel about your child going back to school?**
    - How did you go about it?

14. **How did your child feel about going back to school?**
    - And how did the child find the experience**?**

15. **Did your child have a school visit? What did you think about this?**
    - Did it help the transition?
    - In what way?

16. **Could you tell me about any changes between your child's schooling prior to the cancer diagnosis and the return to school after treatment?**
    - Cognitive? Physical? Social? Psychological?
    - What did you/others do about this?

17. **Was there anything that you can identify that made the transition back to school easier for you and your child?**

<u>SCHOOLING NOW</u>

18. **What is your child like at school now?**
    - Academically? Socially? Physically? Engagement-wise?
    - If repeated a grade, ask more about this

19. **How does your child feel about school now?**

20. **What is the child's attendance like now?**

21. **Does the child participate in any extracurricular activities?**

22. **Are there any ongoing challenges?**

23. **Have you or do you use any support from any non-government agencies, such as The Ronald McDonald Learning Program or the Childhood Cancer Association?**

24. **Is there anything that makes you worry about your child's schooling?**
    - Do you worry about the ability to achieve academically? Socially?

25. **Regarding siblings – have you noticed any school-related changes for any of your other children?**
    - Any changes in attendance, academic achievement, social functioning?

26. **What do you think the communication was like between the hospital, your child's school, and you?**
    - Do you feel as though you all worked together?

27. **Overall, did you feel that schooling was an important aspect of your child's cancer care?**
    -Were you getting this message? From everyone?

28. **Overall, were you satisfied with the opportunities provided by the [children's hospital] to maintain your child's schooling**

29. **Overall, what have been the main challenges relating to schooling – for your child? For you?**
    - Did you feel that you were supported? That these challenges were addressed?

30. **Do you have any suggestions for additions/improvements to school-related programs at the [children's hospital]?**

31. **Do you have any suggestions/advice for other parents related to having a child at school with cancer generally?**
